# L-Arginine supplementation improves the survival of virus-specific CD8^+^ T cells during *in vitro* expansion

**DOI:** 10.1093/immadv/ltag014

**Published:** 2026-09-16

**Authors:** Takahiro Tomoda, Ai Kawana-Tachikawa, Shigeomi Shimizu, Satoshi Takahashi, Hirokazu Kanegane, Takahiro Kamiya, Kumi Inoue, Masatoshi Takagi, Tomohiro Morio

**Affiliations:** Department of Pediatrics and Developmental Biology, Institute of Science Tokyo, Tokyo, Japan; AIDS Research Center, National Institute of Infectious Diseases, Japan Institute for Health Security, Tokyo, Japan; Department of Pathological Cell Biology, Institute of Science Tokyo, Tokyo, Japan; Division of Clinical Precision Research Platform, Institute of Medical Science, The University of Tokyo, Tokyo, Japan; Department of Child Health and Development, Institute of Science Tokyo, Tokyo, Japan; Department of Pediatrics and Developmental Biology, Institute of Science Tokyo, Tokyo, Japan; Laboratory of Immunology and Molecular Medicine, Advanced Research Initiative, Institute of Science Tokyo, Tokyo, Japan; Department of Pediatrics and Developmental Biology, Institute of Science Tokyo, Tokyo, Japan; Laboratory of Immunology and Molecular Medicine, Advanced Research Initiative, Institute of Science Tokyo, Tokyo, Japan

**Keywords:** L-arginine, virus-specific T-cell therapy, apoptosis, cytokine signaling, MCL-1

## Abstract

**Introduction:**

Virus-specific T cell therapy is a promising treatment for life-threatening viral infections following allogeneic hematopoietic stem cell transplantation. However, its efficacy is limited by apoptotic cell loss during *in vitro* expansion and the cryopreservation process.

**Materials and methods:**

Human virus-specific T cells were expanded *in vitro* with interleukin-7 and interleukin-15 in RPMI 1640-based medium with or without L-arginine supplementation, and their survival, phenotype, mitochondrial function, and cytokine signaling were evaluated.

**Results:**

Supplementation of the culture medium with L-arginine enhanced the survival of human virus-specific T cells during *in vitro* expansion. Supplementation of the RPMI 1640-based culture medium with L-arginine reduced apoptosis and was accompanied by increased expression of the anti-apoptotic protein MCL-1 in virus-specific CD8^+^ T cells. In parallel, modest differences in exhaustion- and differentiation-associated markers were observed. Consistent with these survival-associated changes, preservation of mitochondrial membrane potential was concurrently observed in non-apoptotic CD8^+^ T cells. Among several culture-associated factors that may contribute to mitochondrial preservation, L-arginine availability was associated, at least in part, with common gamma-chain cytokine-induced phosphorylation of mammalian target of rapamycin signalling and signal transducer and activator of transcription 5 downstream of interleukin-7 and interleukin-15 during *in vitro* culture.

**Conclusion:**

Together, these findings support L-arginine supplementation as a simple and clinically applicable strategy to enhance the durability of virus-specific T cell products during *in vitro* expansion, as well as cryopreservation and thawing.

## Graphical Abstract

**Graphical Abstract:**
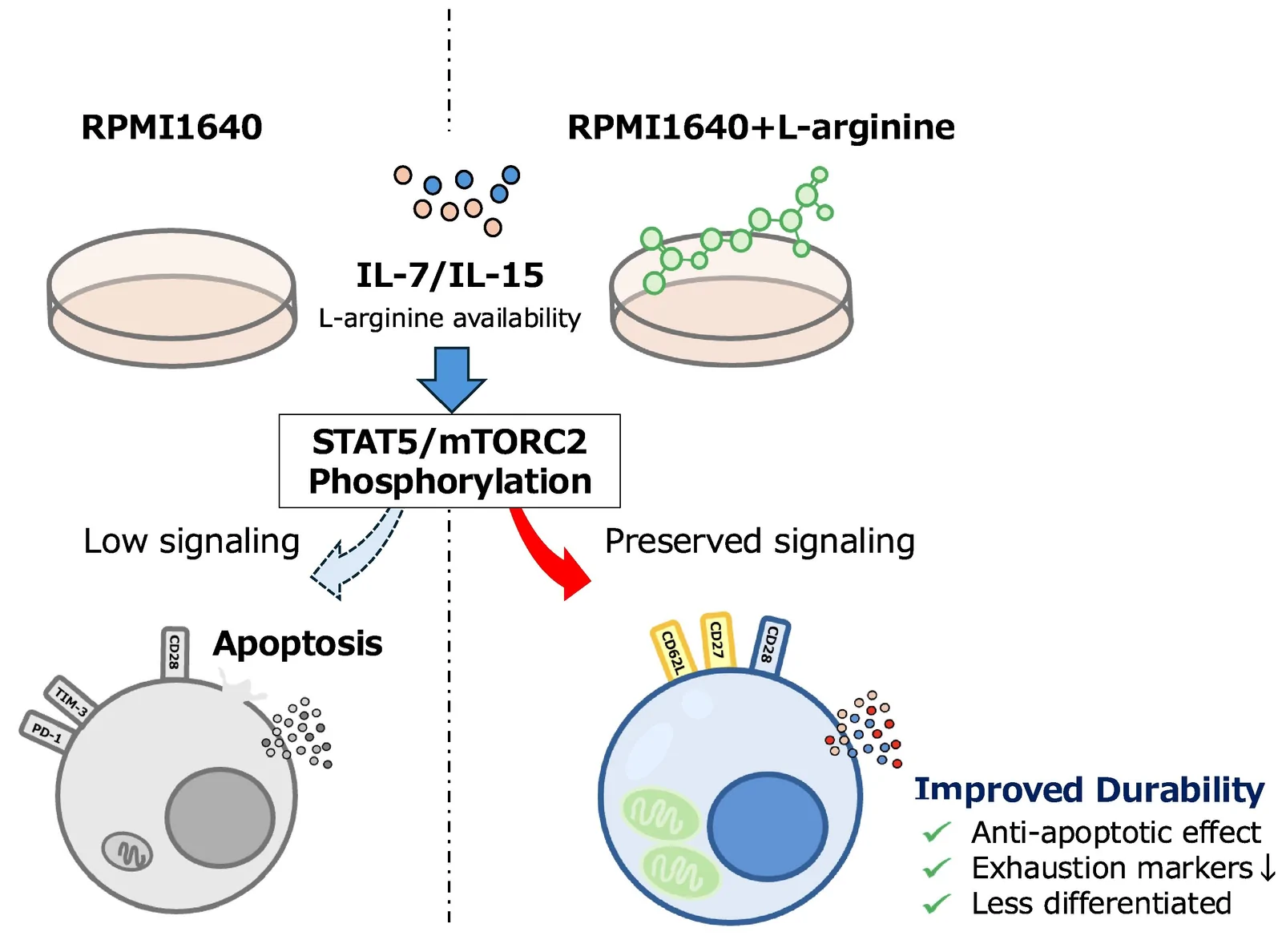

## Introduction

Virus-specific T-cell (VST) therapy has emerged as a promising therapeutic approach for the management of treatment-refractory viral reactivation following hematopoietic stem cell transplantation [1–3]. However, its efficacy is frequently compromised by apoptosis and functional deterioration during *in vitro* expansion and following freeze–thawing [4–7]. These vulnerabilities are closely linked to mitochondrial function [8, 9], prompting the exploration of interventions, such as PD-1 blockade and succinate supplementation to support T-cell function [10, 11].

L-Arginine is a nonessential amino acid that is utilized by multiple immune cell types, including T cells, antigen-presenting cells (APCs), and innate immune cells [12, 13]. It is transported via cationic amino acid transporters and metabolized by arginase 1 and nitric oxide synthase 2, yielding intermediates involved in cellular proliferation and redox regulation [12, 14]. In murine and cellular model systems, L-arginine promotes mitochondrial biogenesis in T cells through nicotinamide adenine dinucleotide (NAD^+^)-linked metabolism [15, 16] and activates nutrient-sensing pathways, including mammalian target of rapamycin complex 1 (mTORC1) and mTORC2 signaling [17–19]. In contrast, evidence in human T cells remains limited. L-Arginine uptake has been reported to enhance the survival of activated naive CD4^+^ T cells via mechanisms independent of canonical mTOR signaling [20]. While its downstream metabolite, spermidine, also supports mitochondrial biogenesis in murine CD8^+^ T cells [21], it has been reported to reduce the survival of activated naive CD4^+^ T cells in a human study [20]. Moreover, excessive nitric oxide (NO), a byproduct of L-arginine metabolism, can impair T-cell function and disrupt pH homeostasis, leading to growth inhibition and apoptosis [12, 22, 23]. Despite these insights, the impact ofL-arginine supplementation on human antigen-experienced, differentiated memory VSTs has not been directly evaluated. This question is particularly relevant because VSTs exposed to chronic antigen stimulation acquire central or effector memory–like phenotypes with metabolic dependencies distinct from those of naive T cells [4, 24, 25].

In this study, we investigated the effect of supplementing RPMI 1640 medium with L-arginine—a simple modification of a widely used culture condition—on the survival and phenotypic profile of human VSTs.

## Materials and methods

### Study participants

Ten healthy adult donors were recruited. VSTs were generated from seven donors and used for the primary analyses, whereas cells from the remaining three donors were used exclusively for D-arginine stereospecific control experiments. Peripheral blood samples were obtained with written informed consent in accordance with institutional guidelines.

### Peptides

Pools of 15-mer overlapping peptides (overlap of 11 amino acids) spanning pp65 and IE1 in cytomegalovirus (CMV); EBNA1, LMP2A, and BZLF1 in Epstein–Barr virus (EBV); Hexon and Penton in human adenovirus (HAdV); VP1 and large T in BK polyomavirus; VP1 and large T in JC polyomavirus (Miltenyi Biotec, Germany); and U54 and U90 in human herpesvirus 6 (JPT, Germany) were used.

### Generation of multivirus-specific T cells

Peripheral blood mononuclear cells (PBMCs) were isolated using Ficoll density–gradient centrifugation. A total of 2 × 10^6^ PBMCs were stimulated with the viral peptides (100 ng/ml each) and cultured in RPMI 1640 (FUJIFILM Wako, Japan) supplemented with 10% heat-inactivated fetal calf serum (Nichirei Bioscience, Japan), 100 U/ml penicillin, and 100 μg/ml streptomycin (Gibco, NY, USA), 2 mM L-glutamine, 10 mM 4-(2-hydroxyethyl)-1-piperazineethanesulfonic acid (HEPES) (Sigma-Aldrich, MO, USA), and 1% sodium pyruvate (Gibco), collectively referred to as R10, in the presence of 10 ng/ml interleukin (IL)-7, 5 ng/ml IL-15 (Miltenyi Biotec), and 20 ng/ml IL-21 (PeproTech, NJ, USA) at 37°C in a humidified atmosphere with 5% CO_2_. The medium was replaced twice per week with R10 supplemented with IL-7 (10 ng/ml) and IL-15 (5 ng/ml). Cells were harvested on Day 15 for downstream analyses and used as multi-VSTs (MVSTs). Cell counts and viability were assessed using acridine orange–propidium iodide (AO/PI) or trypan blue staining with automated cell counters (LUNA-FL and LUNA-II, Logos Biosystems, South Korea).

L-Arginine was added to R10 (basal medium; control), which already contained 1.15 mM L-arginine (FUJIFILM Wako), to reach a final concentration of 4.15 mM (baseline 1.15 mM + 3.0 mM supplementation) in the L-arginine–enriched medium. The supplementation concentration was determined based on a previous study evaluating supraphysiological L-arginine conditions in naive CD4^+^ T cells [20]. For the stereospecific controls, D-arginine (Tokyo Chemical Industry Co., Ltd, Japan) was used.

### Restimulation and functional expansion assays

Cells obtained on Day 15 were restimulated with the viral peptides (100 ng/ml each) in the presence of 10 ng/ml IL-7, 5 ng/ml IL-15, 20 ng/ml IL-21, 20 ng/ml IL-12, 50 ng/ml IL-18 (Miltenyi Biotec), and 50 ng/ml of TL1A (PeproTech). IL-12, IL-18, and TL1A were included to support sufficient cell expansion for downstream analyses under feeder-free restimulation conditions [26]. These cultures were maintained at 37°C in a humidified atmosphere with 5% CO_2_, with regular supplementation of IL-7 (10 ng/ml) and IL-15 (5 ng/ml) for 14 days to obtain sufficient cell numbers for downstream functional analyses. On Day 29, the expanded cells were harvested and analyzed for functional assays.

### Measurement of extracellular pH in media

The pH of media was measured using the SevenCompact S220 pH meter (Mettler Toledo, Switzerland) after 3 h incubation at 37°C and 5% CO_2_ in the absence of cells.

### Flow cytometric analysis

Data were acquired using FACSCanto II, LSRFortessa X-20, and FACSymphony flow cytometers (BD Biosciences). Lymphocytes were gated by forward/side scatter, and dead cells were removed using LIVE/DEAD Fixable Aqua (LIVE/DEAD Aqua) or Fixable Viability Stain 575V (FVS575V) prior to analysis with antibodies. Analyses were performed using FlowJo version 10.7.1 (BD Biosciences). A detailed list of the antibodies and reagents for flow cytometry analysis is shown in Supplementary Table S1.

### Intracellular cytokine staining

MVSTs were incubated for 12 h in cytokine-free R10 (cytokine withdrawal) prior to stimulation. Subsequently, cells were stimulated with or without the viral peptides (100 ng/ml each) in the presence of anti-CD28 (1 μg/ml), anti-CD49d (1 μg/ml), and GolgiStop for 6 h. Cells were washed once with Dulbecco’s phosphate-buffered saline (D-PBS without Ca^2+^ and Mg^2+^, FUJIFILM Wako), and stained with LIVE/DEAD Aqua Dead Cell Stain, followed by surface staining with fluorochrome-conjugated antibodies against surface markers (CD3, CD4, CD8, and CD107a) for 30 min at 4°C. Cells were then fixed and permeabilized with BD Cytofix Fixation Buffer and Perm/Wash buffer (BD Biosciences), respectively, and stained with anti-interferon-gamma (anti-IFN-γ) and antitumor necrosis factor-alpha (anti-TNF-α) antibodies. Polyfunctionality analysis was performed using SPICE and PESTLE software (National Institute of Health, MD, USA).

### Staining for apoptosis markers

Following cytokine withdrawal, MVSTs were stimulated with or without viral peptide pools for 12 h, as described above. Cells were stained with FVS575V, followed by surface staining with antibodies (CD3, CD4, CD8, CD25, CCR7, CD45RA, CD27, CD28, CD62L, PD-1, and TIM-3). For apoptosis analysis, cells were stained with Annexin V, fixed and permeabilized with prewarmed BD Phosflow Fix Buffer I and BD Phosflow Perm/Wash buffer I (BD Biosciences), respectively, and stained intracellularly with antibodies [cleaved caspase-3 (cCasp-3), MCL-1, BCL-2, BCL-xL, and IFN-γ].

### Inhibitor-based apoptosis assays

An MCL-1 inhibitor (AZD5991; Selleck Chemicals, TX, USA) and a BCL-2 inhibitor (ABT-199; Selleck Chemicals) were used for inhibitor experiments. Both inhibitors were dissolved in dimethyl sulfoxide (DMSO; Sigma-Aldrich), with the final DMSO concentration kept below 0.1% in the culture. After thawing and overnight culture in R10, cryopreserved MVSTs (2 × 10^5^ cells/well) were incubated with IL-7 and IL-15 in the presence of AZD5991 or ABT-199 for 24 h at concentrations ranging from 0 to 1000 nM. The 0 nM condition served as the vehicle control and received an equivalent volume of DMSO. To compare inhibitor sensitivity between culture conditions, MVSTs expanded for 15 days under either R10 or L-arginine–enriched conditions were cryopreserved and subsequently thawed. Following thawing, cells were re-cultured under their respective original culture conditions with IL-7 and IL-15 in the presence of AZD5991 or ABT-199 for 24 h. Apoptosis was assessed using the Annexin V-FITC Apoptosis Detection Kit (BioVision, CA, USA).

### Mitochondrial membrane potential

Mitochondrial membrane potential (MMP) was assessed using the MT-1 MitoMP Detection Kit (Dojindo, Japan) following the manufacturer’s instructions. Briefly, cells were incubated with MT-1 dye in RPMI 1640 for 30 min, followed by staining with LIVE/DEAD Aqua and fluorochrome-conjugated antibodies against CD3, CD4, and CD8. After washing, cells were stained with Annexin V antibody.

### Staining for phosphorylated S6, Akt, signal transducer and activator of transcription 5, and inhibitor-based signaling analysis

After thawing and overnight rest in R10, cryopreserved MVSTs were incubated for 1 h in L-arginine–free medium composed of 0.3% (w/v) bovine serum albumin (Sigma-Aldrich), 10 mM HEPES, 1% sodium pyruvate, 5 mM glucose (Otsuka, Japan), 1 mM MgCl_2_ (FUJIFILM Wako), and 100 U/ml penicillin and 100 μg/ml streptomycin in PBS. This simplified buffer system minimized background activation of common γ-chain (γc) cytokine signaling from other amino acids or serum components, thereby enabling accurate assessment of the acute contribution of extracellular L-arginine. After this preincubation, the cells were stained with LIVE/DEAD Aqua and transferred to the L-arginine–free medium with or without 1 mM L-arginine. For acute signaling analysis, cells were stimulated with IL-7 (10 ng/ml) and IL-15 (5 ng/ml) for 15 or 30 min to assess Akt and S6 phosphorylation, or with IL-2 (5 U/ml, Miltenyi Biotec), IL-7 (10 ng/ml), or IL-15 (2.5 ng/ml) for 30 or 60 min to assess signal transducer and activator of transcription 5 (STAT5) phosphorylation. The reduced IL-15 concentration was used to avoid signal saturation. As a positive control for phosphorylation of Akt and S6, cells were stimulated with PMA (50 ng/ml; Sigma-Aldrich) and ionomycin (1 μg/ml; Sigma-Aldrich) for 15 min prior to fixation. Cells were fixed with prewarmed BD Phosflow Fix Buffer I, permeabilized with Phosflow Perm buffer III (BD Biosciences), and stained with antibodies against CD3, CD4, CD8, and intracellular antibodies against pS6 and pAkt, or pSTAT5. For inhibitor-based signaling dissection, cells were cultured under the same L-arginine and cytokine conditions for 48 h and treated with sapanisertib (100 nM; Selleck Chemicals) and/or tofacitinib (50 nM; Selleck Chemicals), with DMSO as vehicle control. Apoptosis and MMP were assessed by Annexin V and MT-1 staining.

### Cryopreservation and thawing

MVSTs were cryopreserved in Cellbanker (ZENOAQ, Japan) using a BICELL freezing container (Nihon Freezer, Japan) at −80°C overnight and subsequently transferred to liquid nitrogen until use. Frozen cells were rapidly thawed at 37°C and cultured overnight in R10 medium before downstream analyses.

### Statistical analyses

The Wilcoxon matched-pairs signed-rank test was used for comparisons. *P* values of <.05 were considered statistically significant. Statistical significance in figures is indicated as follows: *P < .05. Nonsignificant differences are indicated as ns. GraphPad Prism v10.3.6 (GraphPad Inc., CA, USA) was used for all statistical analyses.

## Results

### Generation of multivirus-specific T cells

PBMCs from 7 healthy donors were stimulated with overlapping peptides derived from multiple viral antigens and cultured in R10 with IL-7 and IL-15 for 15 days to generate MVSTs. The cells were expanded 4- to 39-fold (median: 8-fold) during the 2-week culture period (data not shown). Most cells were CD3^+^, and the CD8^+^ subset was dominant (Supplementary Fig. S1a and b). The frequency of virus-specific CD8^+^ T cells was 34.1% (median, range 5.8%–52.6%) with CMV-specific being dominant, followed by EBV- and HAdV-specific cells (Supplementary Fig. S1c).

### Induction of apoptosis during the generation of multivirus-specific T cells

**Figure 1:**
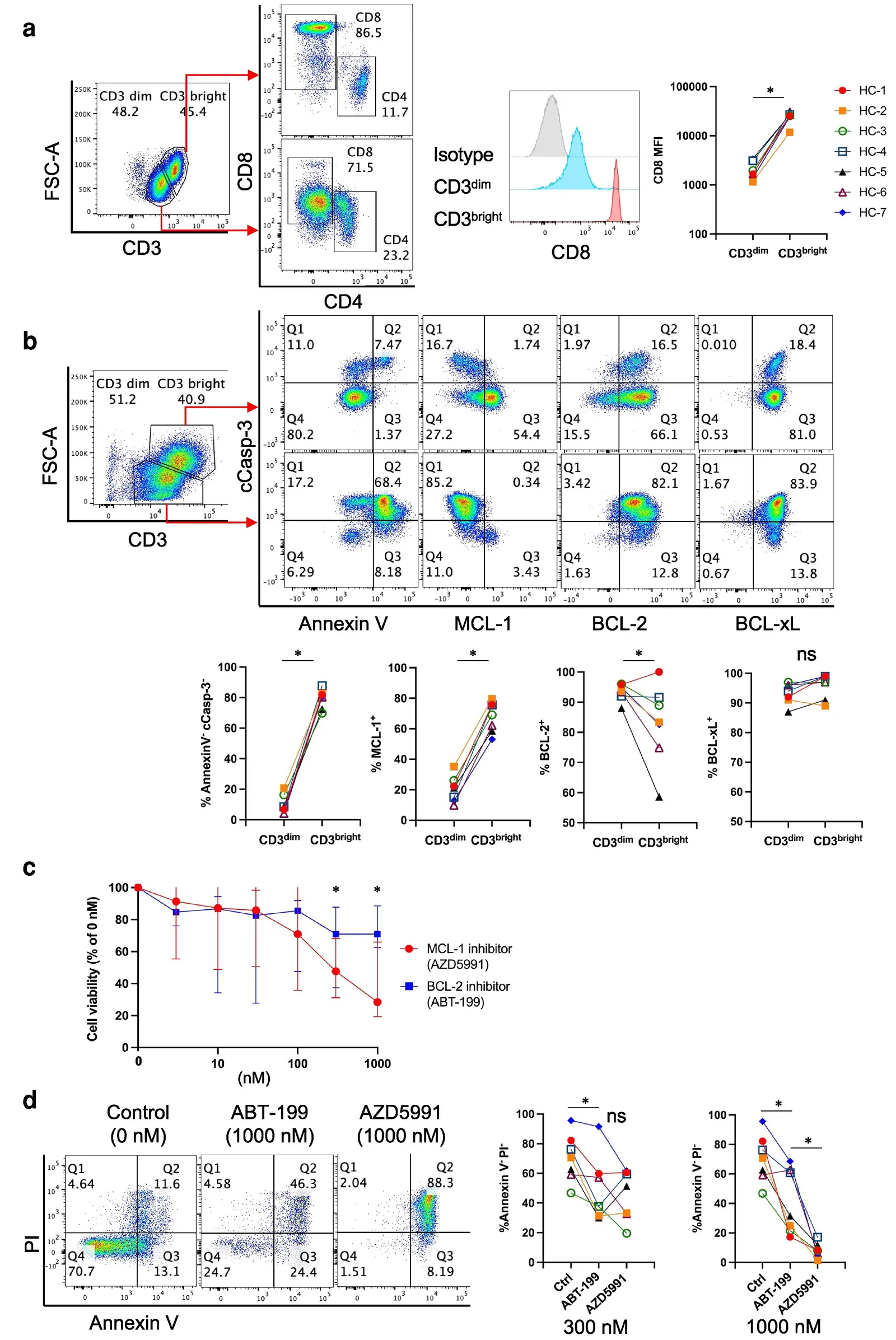
Analysis of apoptosis and the functional role of MCL-1 in MVSTs. (a) Representative flow cytometric plots and the MFI of CD8 in CD3^bright^ and CD3^dim^ T cells in MVSTs. The gating strategy is shown in Supplementary Fig. S1a. (b) Representative flow cytometric plots and expression of apoptotic markers (Annexin V and cCasp-3) and antiapoptotic markers (MCL-1, BCL-2, and BCL-xL) in CD3^dim^ and CD3^bright^ subsets of T cells. (c) MVSTs were cultured in the presence of IL-7 and IL-15 and treated with increasing concentrations of AZD5991 or ABT-199 for 24 h. Viable cell numbers were determined by trypan blue exclusion. (d) Annexin V and PI staining of CD3^+^ T cells following treatment with AZD5991 or ABT-199 for 24 h in the presence of IL-7 and IL-15. The gating strategy is shown in Supplementary Fig. S1d. Each dot represents an individual donor (biological replicate; *n* = 7, as indicated). Data in (c) are shown as median interquartile range; all other panels are presented as individual donor values. Data were obtained from one independent experiment per donor. Statistical comparisons were performed using a paired Wilcoxon matched-pairs signed-rank test. Statistical significance is indicated as follows: ns, not significant; *P < .05.

The CD3^+^ cells in the MVSTs were distinctly separated based on CD3 expression levels into CD3^dim^ (lower CD3 expression) and CD3^bright^ (higher CD3 expression) subsets (Fig. 1a, *P* = .0156). When the cells were further analyzed based on CD4 and CD8 expression, both CD8 and CD4 expression were significantly lower in the CD3^dim^ subset compared with the CD3^bright^ subset (Fig. 1a). Given that CD3 and CD8 expression were downregulated on apoptotic T cells [27], the apoptotic status of MVSTs was assessed. The majority of CD3^dim^ T cells showed markedly higher Annexin V and/or cCasp-3 and lower MCL-1 (Fig. 1b, *P* = .0156), indicating that CD3^dim^ cells were undergoing apoptosis. Notably, cCasp-3 and MCL-1 expression were mutually exclusive. BCL-2 expression patterns varied between donors, but were consistently higher in CD3^dim^ cells despite elevated cCasp-3 levels, and BCL-xL expression did not differ between the subsets (Fig. 1b, *P* = .0312). These data suggest that MCL-1 plays an important role in the antiapoptotic effects in MVSTs. To assess whether MCL-1 functionally contributes to T-cell survival in these culture conditions, the MVSTs were treated with inhibitors targeting either MCL-1 (AZD5991) or BCL-2 (ABT-199) in the presence of IL-7 and IL-15, consistent with the cytokine conditions used during *in vitro* expansion. When cell viability of the MVSTs was assessed after 24 h culture under these inhibitors, the viable cell numbers were significantly decreased with AZD5991 but not ABT-199 treatment in a dose-dependent manner (Fig. 1c, *P* = .0156). Consistently, AZD5991 markedly reduced the frequency of viable cells (Annexin V^−^ and PI^−^) (Fig. 1d and Supplementary Fig. S1d, *P* = .0156). These results suggest that MCL-1 plays a central role in maintaining the viability of the MVSTs in the culture condition.

### Effect of L-arginine supplementation on apoptosis resistance in multivirus-specific T cells

**Figure 2:**
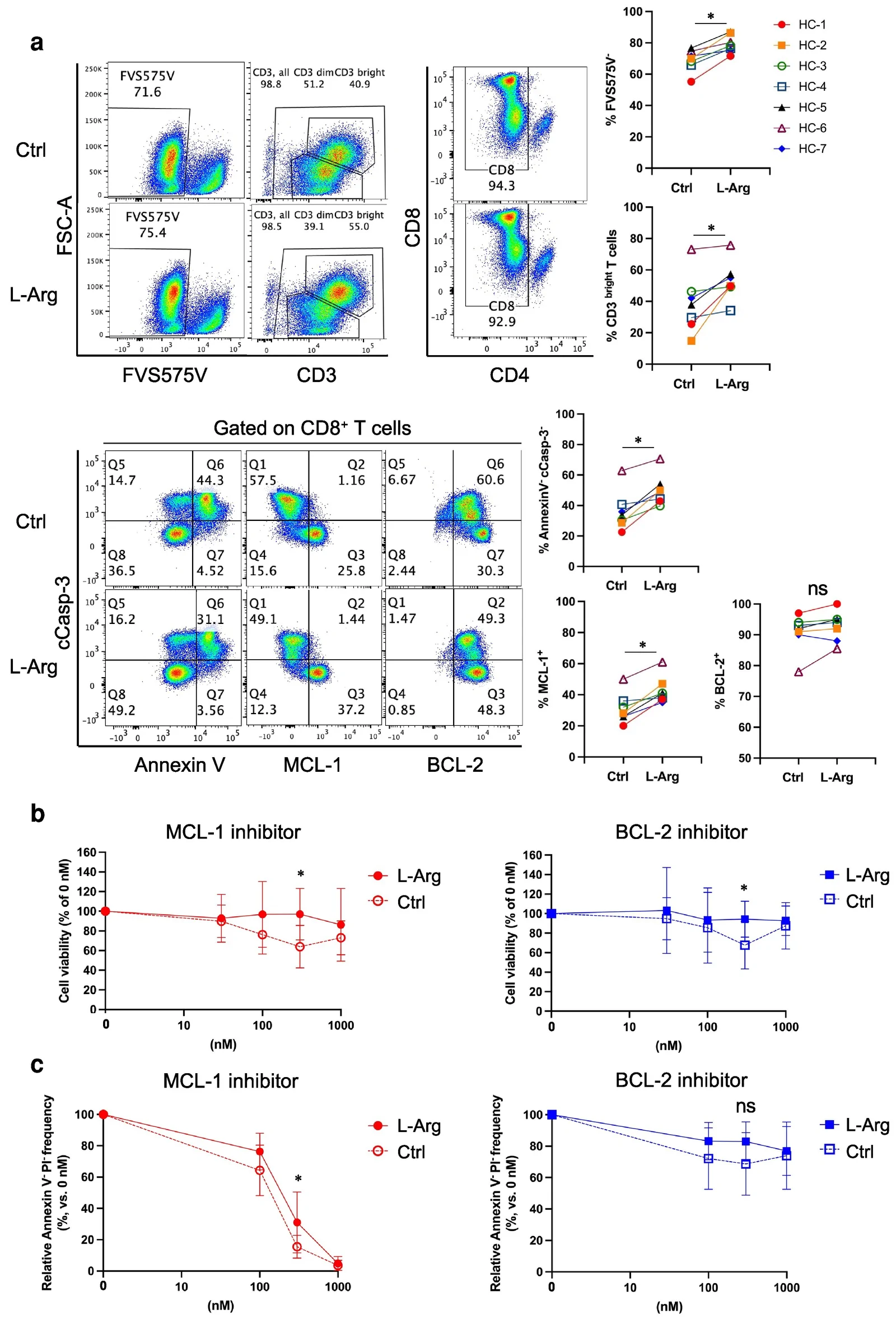
Effects of L-arginine supplementation on apoptosis-related phenotypes and inhibitor sensitivity in MVSTs. (a–c) Apoptotic and MCL-1 inhibition analyses of CD8^+^ T cells within MVSTs cultured under R10 (Ctrl) or L-arginine–enriched conditions (L-Arg). (a) Representative flow cytometric plots and frequencies of viable cells (FVS575V-negative), apoptotic markers (Annexin V and cCasp-3) and antiapoptotic markers (MCL-1 and BCL-2; *n* = 7). (b) Relative viable cell number of MVSTs cultured under R10 or L-arginine–enriched conditions in the presence of IL-7 and IL-15 with either MCL-1 inhibitor AZD5991 (left) or BCL-2 inhibitor ABT-199 (right) for 24 h. To evaluate relative sensitivity to MCL-1 or BCL-2 inhibition independent of baseline differences in viability between culture conditions, values at each inhibitor concentration were normalized to the corresponding 0 nM control within each culture condition. Absolute viable cell numbers before normalization are shown in Supplementary Fig. S2c. (c) Relative frequencies of Annexin V^−^ PI^−^ CD8^+^ T cells in MVSTs cultured under R10 or L-arginine–enriched conditions in the presence of IL-7 and IL-15 with either AZD5991 (left) or ABT-199 (right) for 24 h. To evaluate relative sensitivity to MCL-1 or BCL-2 inhibition independent of baseline differences in viability between culture conditions, values at each inhibitor concentration were normalized to the corresponding 0 nM control within each culture condition. Absolute frequencies before normalization are shown in Supplementary Fig. S2d. Data in (b) and (c) are shown as median interquartile range; all other panels are presented as individual donor values. Each dot represents an individual donor (biological replicate; *n* = 7). Data were obtained from one independent experiment per donor. Statistical comparisons were performed using a paired Wilcoxon matched-pairs signed-rank test. Statistical significance is indicated as follows: ns, not significant; *P < .05.

To evaluate the effects of L-arginine supplementation during the MVST expansion, PBMCs were stimulated with the viral peptides in either R10 or L-arginine–enriched R10 under the same cytokine condition used in the previous experiments. Cell proliferation was comparable between the two conditions (Supplementary Fig. S2a). Nevertheless, the frequencies of viable cells (FVS575V-negative), CD3^bright^ T cells, nonapoptotic CD8^+^ T cells (cCasp-3^−^ and Annexin V^−^), and MCL-1 expression were significantly higher under the L-arginine–enriched condition (Fig. 2a, *P* = .0156 for all comparisons). In contrast, the expression of BCL-2 did not differ between the two conditions. Given the selective increase in MCL-1 expression under the L-arginine–enriched condition, we next investigated whether L-arginine supplementation altered sensitivity to apoptosis induced by MCL-1 or BCL-2 inhibition. Equal numbers of viable MVSTs were cultured for 24 h in R10 or L-arginine–enriched medium supplemented with IL-7 and IL-15 in the presence of increasing concentrations of each inhibitor. Under MCL-1 inhibition, MVSTs cultured under the L-arginine–enriched condition exhibited significantly higher viable cell numbers at 100 and 300 nM AZD5991 treatment compared with those cultured under the control condition (Supplementary Fig. S2c, left, *P* = .0312 and *P* = .0156). Because viable numbers were already higher in the L-arginine group under vehicle control conditions (0 nM), values at each inhibitor concentration were normalized to the corresponding 0 nM control within each condition to evaluate relative sensitivity to inhibitor-induced cell loss. Using this approach, MVSTs cultured under the L-arginine–enriched condition retained significantly higher viable cell numbers and frequencies of Annexin V^−^ PI^−^ CD8^+^ T cells at 300 nM AZD5991 treatment (Fig. 2b and c, left panels, *P* = .0156 for both analyses). Frequencies of Annexin V^−^ PI^−^ CD8^+^ T cells were also significantly higher under the L-arginine–enriched condition before normalization (Supplementary Fig. S2d, left panel, *P* = .0312). In contrast, under BCL-2 inhibition, the protective effects of L-arginine supplementation were less consistent. Although higher viable cell numbers and frequencies of Annexin V^−^ PI^−^ CD8^+^ T cells were observed at several ABT-199 concentrations before normalization (Supplementary Fig. S2c and d, right panels, *P* = .0312, *P* = .0156, *P* = .0156, and *P* = .0469), these differences became less pronounced after normalization to the corresponding 0 nM controls (Fig. 2b and c, *P* = .0469). Because L-arginine supplementation increased the extracellular pH of the culture medium and it is known that an elevated pH suppresses cell proliferation and induces apoptosis [23], the same experiments were performed using D-arginine, the optical isomer of L-arginine. Despite comparable extracellular pH conditions (Supplementary Fig. S2e), D-arginine supplementation did not show the antiapoptotic effects observed under the L-arginine–enriched condition (Supplementary Fig. S2f, *P* = .0156), suggesting that the antiapoptotic effect is not solely attributable to extracellular pH change. Collectively, these findings suggest that L-arginine supplementation enhances apoptosis resistance in MVSTs and partially preserves survival under conditions of MCL-1 inhibition.

### Effect of L-arginine supplementation on mitochondrial integrity and phenotypic characteristics of multivirus-specific T cells

**Figure 3:**
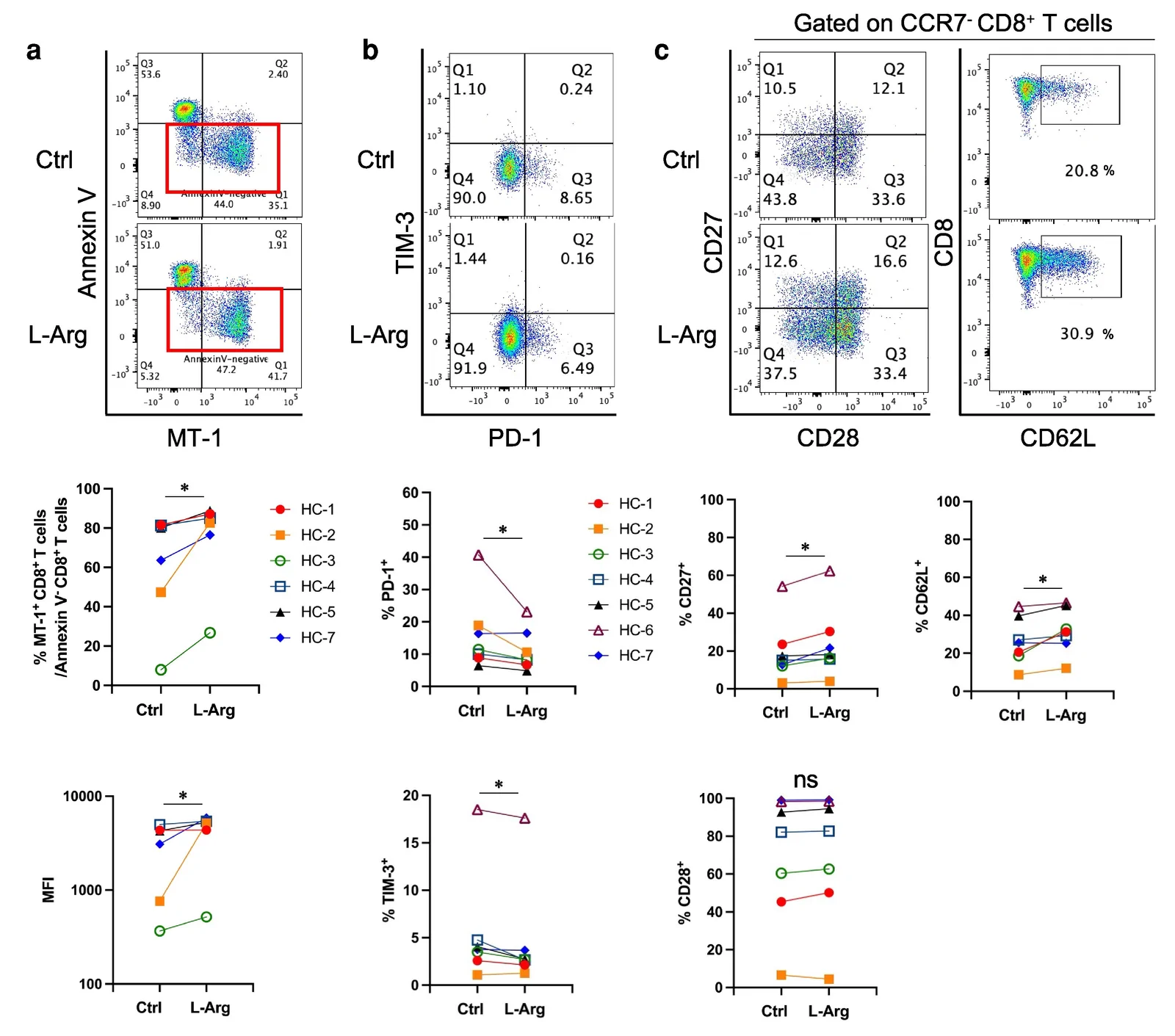
Effects of L-arginine supplementation on mitochondrial membrane potential, exhaustion, and differentiation phenotypes in CD8^+^ T cells within MVSTs. (a) Representative flow cytometric plots and frequency and MFI of MT-1^+^ cells among Annexin V^−^ CD8^+^ T cells (*n* = 6 due to limited cell numbers in one donor). The gating strategy is shown in Supplementary Fig. S3a. (b) Expression of exhaustion markers (PD-1 and TIM-3) in CD8^+^ T cells (*n* = 7). The gating strategy is shown in Supplementary Fig. S3b. (c) Expression of differentiation markers (CD27, CD28, and CD62L) in CCR7^−^ CD8^+^ T cells (*n* = 7). Memory T-cell subset classification based on CCR7 and CD45RA expression is shown in Supplementary Fig. S3c. Each dot represents an individual donor (biological replicate; *n* = 7). Data were obtained from one independent experiment per donor. Statistical comparisons were performed using a paired Wilcoxon matched-pairs signed-rank test. Statistical significance is indicated as follows: ns, not significant; *P < .05.

MCL-1 maintains mitochondrial integrity and prevents intrinsic apoptotic signaling [28]. To further characterize cellular integrity in the context of enhanced survival, MMP, an indicator of mitochondrial integrity [29], was assessed using MT-1 staining. Both the frequency and median fluorescence intensity (MFI) of MT-1-positive cells among the Annexin V^−^ CD8^+^ T cells were significantly higher under the L-arginine–enriched condition (Fig. 3a and Supplementary Fig. S3a, *P* = .0312). In addition to these survival-associated changes, CD8^+^ T cells cultured under the L-arginine–enriched condition exhibited slightly but significantly lower expression of PD-1 and TIM-3 (Fig. 3b and Supplementary Fig. S3b, *P* = .0156 and *P* = .0469). Although the overall distribution of memory phenotypes defined by CCR7 and CD45RA was not altered (Supplementary Fig. S3c), increased expression of CD27 and CD62L was observed within the CCR7^−^ CD8^+^ T cells (Fig. 3c and Supplementary Fig. S3b, *P* = .0156). These data suggest that L-arginine supplementation preserved mitochondrial integrity while modestly modulating exhaustion- and differentiation-associated phenotypes in CD8^+^ T cells within MVSTs.

### Effects of L-arginine supplementation on virus-specific CD8^+^ T cells

**Figure 4:**
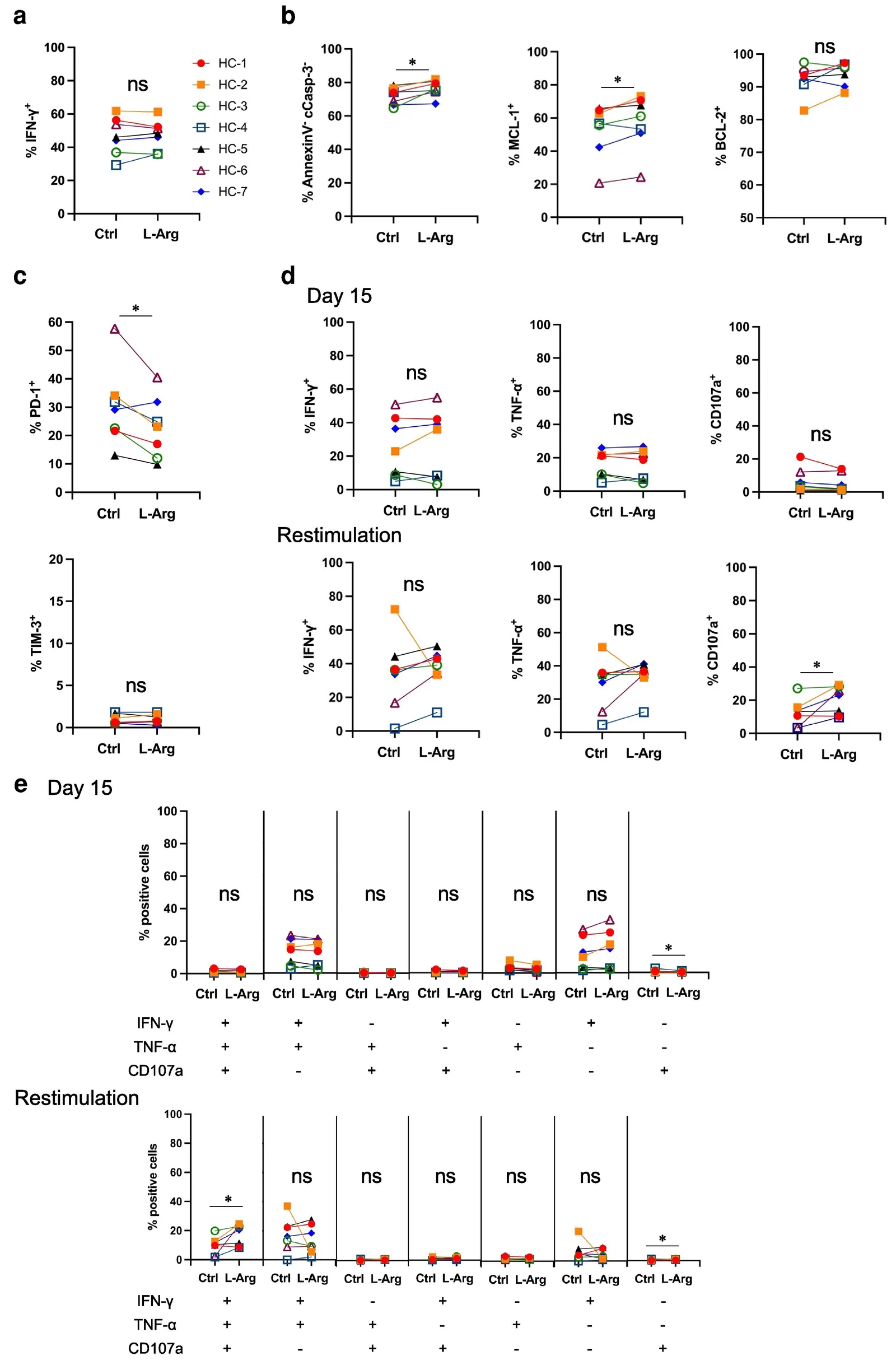
Effects of L-arginine supplementation on apoptosis, exhaustion, and antigen-specific effector function in virus-specific CD8^+^ T cells. (a) Frequencies of virus-specific (IFN-γ–expressing) CD8^+^ T cells. (b) Expression of apoptotic markers (Annexin V and cCasp-3) and antiapoptotic markers (MCL-1 and BCL-2) in virus-specific CD8^+^ T cells. Representative flow cytometric plots are shown in Supplementary Fig. S4a. (c) Expression of exhaustion markers (PD-1 and TIM-3) in virus-specific CD8^+^ T cells. The gating strategy is shown in Supplementary Fig. S4b. Representative flow cytometric plots are shown in Supplementary Fig. S4c. (d) Frequencies of IFN-γ^+^, TNF-α^+^, and CD107a^+^ CD8^+^ T cells following peptide stimulation at Day 15 and after restimulation followed by an additional 14-day culture period under control and L-arginine–enriched conditions. Representative flow cytometric plots are shown in Supplementary Fig. S4d. (e) Polyfunctionality analysis of CD8^+^ T cells following peptide stimulation at Day 15 and after restimulation followed by an additional 14-day culture period under both conditions. Each dot represents an individual donor (biological replicate; *n* = 7). Data were obtained from one independent experiment per donor. Statistical comparisons were performed using a paired Wilcoxon matched-pairs signed-rank test. Statistical significance is indicated as follows: ns, not significant; *P < .05.

Next, we investigated the effect of L-arginine supplementation on virus-specific CD8^+^ T cells within the MVSTs, identified based on IFN-γ production following viral peptide stimulation. The frequencies of IFN-γ^+^ virus-specific CD8^+^ T cells were comparable between the control and L-arginine–enriched conditions at Day 15 (Fig. 4a). We first assessed the survival phenotype of virus-specific CD8^+^ T cells. The proportions of nonapoptotic (cCasp-3^−^ and Annexin V^−^) and MCL-1–expressing cells were significantly higher under the L-arginine–enriched condition, whereas no significant difference was observed in BCL-2 expression, consistent with the findings in the whole MVST population (Fig. 4b and Supplementary Fig. S4a, *P* = .0156 and *P* = .0469). PD-1 expression was also significantly lower under the L-arginine–enriched condition (Fig. 4c, Supplementary Fig. S4b and c, *P* = .0312). We next evaluated antigen-specific effector function. Although no significant differences were observed in IFN-γ, TNF-α, or CD107a, expression at Day 15 (Fig. 4d and Supplementary Fig. S4d), polyfunctionality analysis demonstrated that the proportion of CD107a single-positive cells was significantly lower under the L-arginine–enriched condition (Fig. 4e, *P* = .0312). In contrast, following restimulation and an additional 14-day culture period, CD107a expression became significantly higher under the L-arginine–enriched condition (Fig. 4d, *P* = .0469). Polyfunctionality analysis further demonstrated increased proportions of both CD107a single-positive cells and highly polyfunctional cells simultaneously expressing IFN-γ, TNF-α, and CD107a after restimulation (Fig. 4e, *P* = .0469). These findings suggest that L-arginine supplementation supports the maintenance of antigen-specific effector function following repeated stimulation. Together, these findings indicate that L-arginine supplementation improves survival and supports the maintenance of antigen-specific effector function in virus-specific CD8^+^ T cells following restimulation.

### Enhancement of postcryopreservation resilience in virus-specific CD8^+^ T cells by L-arginine

**Figure 5:**
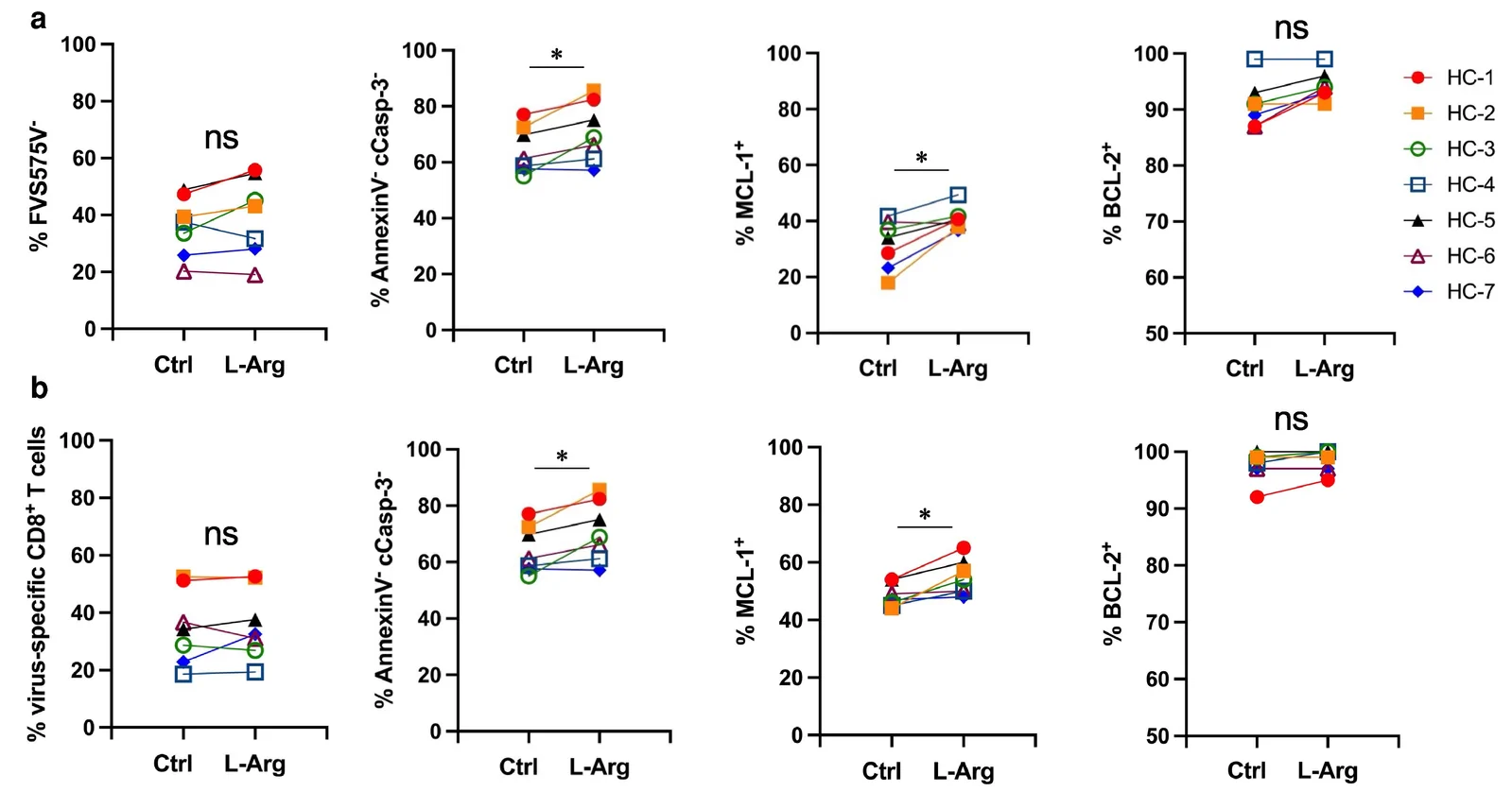
Effects of L-arginine supplementation on cell viability and apoptosis after cryopreservation and thawing. (a) Frequencies of viable cells (FVS575V-negative cells), nonapoptotic cells (Annexin V^−^ and cCasp-3^−^ cells), and CD8^+^ T cells expressing MCL-1 or BCL-2 following cryopreservation and thawing under R10 or L-arginine–enriched conditions. (b) Frequencies of nonapoptotic cells and MCL-1– or BCL-2–expressing virus-specific CD8^+^ T cells after cryopreservation and thawing under the two conditions. Each dot represents an individual donor (biological replicate; *n* = 7). Data were obtained from one independent experiment per donor. Statistical comparisons were performed using a paired Wilcoxon matched-pairs signed-rank test. Statistical significance is indicated as follows: ns, not significant; *P < .05.

To assess the effect of L-arginine supplementation on the quality of virus-specific CD8^+^ T cells after freezing and thawing, cryopreserved MVSTs were thawed and cultured in R10 for 12 h for the analysis. The proportions of nonapoptotic (cCasp-3^−^ and Annexin V^−^) and MCL-1–expressing cells were significantly higher in both the MVSTs (Fig. 5a, *P* = .0469 and *P* = .0312) and virus-specific CD8^+^ T cells in the MVSTs (Fig. 5b, *P* = .0312 and *P* = .0156) cultured under the L-arginine–enriched condition than under the control condition. These findings indicate that the antiapoptotic effects of L-arginine persist in MVSTs even after freezing and thawing.

### Involvement of mammalian target of rapamycin complex 1/2 and signal transducer and activator of transcription 5 signaling in the antiapoptotic effects of L-arginine during multivirus-specific T-cell culture

**Figure 6:**
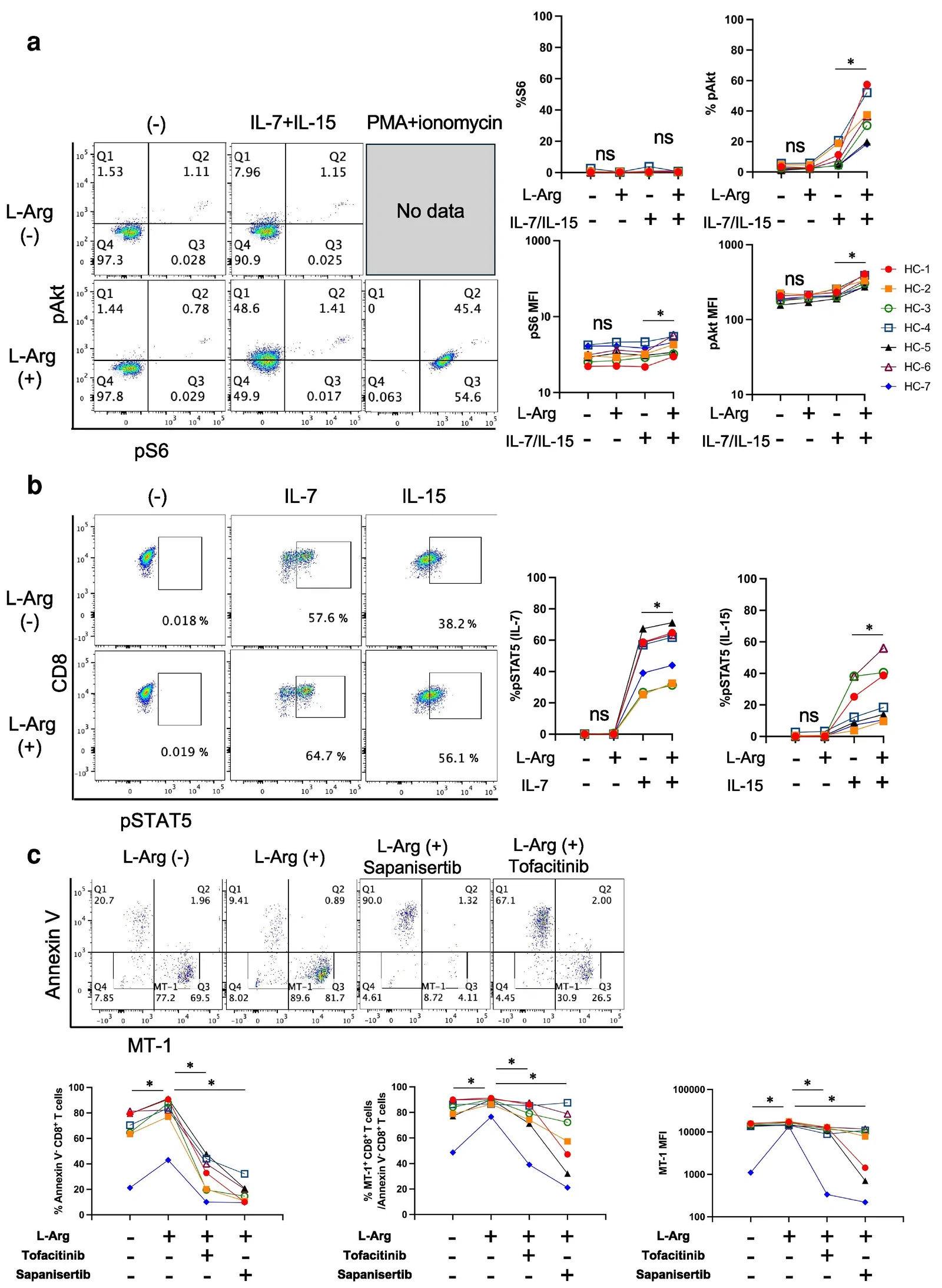
Effects of L-arginine on mTOR and STAT5 signaling in MVSTs.. (a) Representative flow cytometric plots and the frequency and MFI of phosphorylated S6 and Akt in CD8^+^ T cells following stimulation with IL-7 (10 ng/ml) and IL-15 (5 ng/ml) in the presence or absence of 1 mM L-arginine. Cells were fixed at 15 min after stimulation. PMA (50 ng/ml)/ionomycin (1 μg/ml)–stimulated cells were included as positive controls. The gating strategy is shown in Supplementary Fig. S5a. Analysis at 30 min after stimulation is shown in Supplementary Fig. S5b. (b) Representative flow cytometric plots and the frequency of phosphorylated STAT5 in CD8^+^ T cells following stimulation with IL-7 (10 ng/ml) or IL-15 (2.5 ng/ml) in the presence or absence of 1 mM L-arginine for 30 min. Analysis at 60 min after stimulation is shown in Supplementary Fig. S6c. (c) Frequencies of Annexin V^−^ CD8^+^ T cells in MVSTs, and frequencies and MFI of MT-1^+^ cells among Annexin V^−^ CD8^+^ T cells in MVSTs cultured with or without L-arginine under IL-7 and IL-15 stimulation in the presence of sapanisertib or tofacitinib for 48 h. Combined inhibitor treatment was not evaluated because of markedly reduced cell numbers. Each dot represents an individual donor (biological replicate; *n* = 7). Data were obtained from one independent experiment per donor. Statistical comparisons were performed using a paired Wilcoxon matched-pairs signed-rank test. Statistical significance is indicated as follows: ns, not significant; *P < .05.

The PI3K–Akt–mTOR axis plays a critical role in regulating cell survival and proliferation, and L-arginine has been reported to function as an upstream regulator of mTORC1 signaling [17]. In addition, IL-7 and IL-15 promote T-cell survival through STAT5-mediated induction of antiapoptotic molecules, including MCL-1 [30]. To investigate the signaling mechanisms underlying the L-arginine–induced antiapoptotic effect, we first examined Akt and S6 phosphorylation following short-term IL-7/IL-15 stimulation. To eliminate extracellular L-arginine derived from both RPMI 1640 medium and serum, cells were cultured in serum-free and L-arginine–free medium for 1 h before stimulation. Following IL-7/IL-15 stimulation, both the pAkt-positive cells and pAkt MFI were significantly higher under the L-arginine–supplemented condition compared with the L-arginine–free condition at 15 min (Fig. 6a and Supplementary Fig. S5a, *P* = .0156 for both analyses). Increased pAkt expression remained detectable at 30 min (Supplementary Fig. S5b, *P* = .0078 and *P* = .0156 for frequency and MFI, respectively). In parallel, pS6 MFI was significantly higher at 15 min under the L-arginine–supplemented condition (Fig. 6a, *P* = .0156), although this difference was not sustained at 30 min (Supplementary Fig. S5b). Importantly, L-arginine supplementation alone, in the absence of cytokine stimulation, did not induce Akt or S6 phosphorylation (Fig. 6a and Supplementary Fig. S5b). These findings suggest that L-arginine supplementation enhances cytokine-responsive Akt signaling, with only limited and transient enhancement of S6 phosphorylation.

MVSTs maintained under the L-arginine–enriched condition exhibited a significantly higher frequency of CD25^+^ CD8^+^ T cells than those maintained under R10 (Supplementary Fig. S6a, *P* = .0156). Previous studies have reported that IL-7 and IL-15 maintain CD25 expression in T cells through STAT5 signaling [31]. In contrast, NO, a downstream metabolite of L-arginine, has been reported to suppress CD25 expression and IL-2–induced STAT5 phosphorylation [12, 32]. We therefore investigated whether L-arginine supplementation altered cytokine-induced STAT5 phosphorylation. STAT5 phosphorylation induced by IL-7 or IL-15 at 30 min was significantly enhanced in the presence of L-arginine compared with its absence (Fig. 6b, *P* = .0156 for both cytokines). Similar enhancement was observed following IL-2 stimulation (Supplementary Fig. S6b, *P* = .0156). Enhanced STAT5 phosphorylation after IL-15 stimulation remained detectable at 60 min (Supplementary Fig. S6c, *P* = .0156), whereas the differences observed following IL-2 or IL-7 stimulation at 30 min were no longer significant at 60 min (Supplementary Fig. S6c). Notably, L-arginine supplementation alone, in the absence of cytokine stimulation, did not induce STAT5 phosphorylation, with the frequency of pSTAT5-positive cells remaining at zero (Fig. 6b, Supplementary Fig. S6b and c). These findings suggest that L-arginine supplementation potentiates cytokine-responsive STAT5 signaling rather than directly activating STAT5 pathways. Importantly, these observed differences in Akt, S6, and STAT5 phosphorylation were unlikely to be secondary to apoptosis or mitochondrial dysfunction, as short-term L-arginine deprivation did not significantly alter Annexin V staining or MMP (Supplementary Fig. S6d).

To determine whether mTOR- and STAT5-associated signaling pathways contribute to the pro-survival effects of L-arginine supplementation, we performed pharmacological inhibition experiments under the L-arginine–supplemented conditions. Because these signaling pathways are partially interconnected, combined inhibition was also evaluated. After 1 h preculture in L-arginine–free medium, MVSTs were cultured for 48 h with or without L-arginine in the presence of IL-7 and IL-15 together with sapanisertib, a dual mTORC1/2 inhibitor [33], tofacitinib, a JAK1/JAK3 inhibitor that suppresses cytokine-induced STAT5 phosphorylation [34], or both inhibitors in combination. Compared with the L-arginine–free condition, MVSTs cultured in the presence of L-arginine exhibited increased viable cell numbers (Supplementary Fig. S6e, *P* = .0469). This L-arginine–mediated survival advantage was attenuated by treatment with either sapanisertib or tofacitinib and was further reduced by combined inhibition of both pathways (Supplementary Fig. S6e, *P* = .0156), suggesting that mTOR- and STAT5-associated signaling contribute to the survival-promoting effects of L-arginine. Consistent with these findings, the L-arginine–associated increases in Annexin V− cells and in the frequency and MFI of MT-1^+^ cells within Annexin V− CD8^+^ T cells were markedly reduced by either mTORC1/2 or STAT5 pathway inhibition under L-arginine–supplemented conditions (Fig. 6c, *P* = .0156). Combined inhibitor treatment was not evaluated in these flow cytometric analyses because dual inhibition resulted in markedly reduced cell numbers. Collectively, these findings suggest that the pro-survival effects and mitochondrial–protective effects of L-arginine under γc cytokine stimulation are mediated, at least in part, through mTOR- and STAT5-associated signaling pathways.

## Discussion

The present study demonstrates that simple supplementation of RPMI 1640 with L-arginine consistently improves the survival of MVSTs under IL-7– and IL-15–driven expansion conditions. L-Arginine supplementation reduced apoptotic markers, and these benefits persisted after cryopreservation and thawing. These findings directly address a key manufacturing bottleneck in VST production, namely the limited durability of expanded T-cell products under clinically relevant culture and storage conditions.

A central observation of this study is the pivotal role of the antiapoptotic protein MCL-1 in maintaining VST viability. MCL-1 is a key regulator of T-cell survival, particularly under cytokine-dependent and stress-prone conditions [35, 36]. Although T-cell survival is controlled by a network of BCL-2 family members, including BCL-2 and BCL-xL, which can partially compensate for reduced MCL-1 availability [37], our findings indicate a strong functional dependence on MCL-1 in VSTs expanded under γc cytokine–driven conditions. Beyond its canonical antiapoptotic function, MCL-1 has been implicated in supporting mitochondrial homeostasis, underscoring its importance at the interface between mitochondrial stability and cell survival [28].

Given that L-arginine is utilized by multiple immune cell types and can influence cellular function at different stages of immune responses [12, 13], including during antigen stimulation as well as during cytokine-driven culture, the biological effects of L-arginine availability are likely multifaceted and context-dependent. Although the effects of L-arginine on APCs were not examined in this study, we observed antiapoptotic effects of L-arginine not only in antigen-experienced VSTs but also across bulk MVSTs that include nonantigen-specific T cells. These findings suggest that the survival benefit conferred by L-arginine is not restricted to antigen-dependent signaling. Therefore, in the present study, we focused our analysis on cytokine-mediated signaling pathways during *in vitro* culture under conditions that do not involve antigen presentation by APCs. γc cytokines such as IL-7 and IL-15 are central regulators of T-cell survival through activation of STAT5 and mTOR signaling pathways [30, 38, 39]. In the context of cytokine-driven *in vitro* expansion, our data indicate that L-arginine availability is associated with the efficiency of γc cytokine–driven signaling, rather than directly promoting anabolic activation. Accordingly, the differences observed between R10 and L-arginine–supplemented cultures may, at least in part, reflect variations in L-arginine availability during the culture phase, although additional contributing factors cannot be excluded.

Preservation of mTORC2 signaling has been implicated in supporting mitochondrial integrity and survival, regulating forkhead box protein O1 (FOXO1), thereby limiting apoptosis, exhaustion, and terminal differentiation during chronic antigen stimulation [40, 41]. In contrast, excessive mTORC1 activation has been associated with loss of mitochondrial integrity in T cells [10]. It is therefore plausible that L-arginine favors mTORC2-linked survival signaling over mTORC1 signaling in VSTs, thereby supporting MCL-1–associated mitochondrial stability without evidence of excessive mTORC1 activation.

IL-7 and IL-15—both of which signal through STAT5—represent key cytokines in our culture system [42]. Our findings indicate that L-arginine availability supports efficient STAT5 signaling downstream of IL-7 and IL-15 during *in vitro* expansion. This preservation of IL-7/IL-15–mediated STAT5 activity may contribute to the enhanced survival observed in L-arginine–supplemented cultures and may be associated with reduced expression of exhaustion-related markers, as well as the maintenance of CD27 and CD62L expression [42–45]. Together, these findings suggest a mechanistic link between L-arginine availability, cytokine responsiveness, and T-cell persistence.

As T cells undergo prolonged stimulation and differentiation, they become increasingly susceptible to mitochondrial dysfunction and apoptotic stress [46, 47]. Although the MVSTs analyzed in this study predominantly exhibited differentiated memory phenotypes, L-arginine supplementation conferred survival and mitochondrial integrity benefits across these subsets. This suggests that the protective effects of L-arginine are not restricted to early memory T cells but may extend to more differentiated VST populations that are vulnerable to culture-induced stress. Consistent with preserved cellular fitness, L-arginine–treated VSTs demonstrated enhanced functional capacity following repeated stimulation. Restimulated cells exhibited increased CD107a expression together with enhanced polyfunctionality under L-arginine–enriched conditions. These findings suggest that L-arginine supplementation supports the maintenance of antigen-specific effector function during prolonged culture and repeated stimulation, potentially through suppression of excessive differentiation and/or exhaustion [20]. Because L-arginine supplementation was maintained throughout both the initial expansion and postrestimulation phases, the relative contribution of each phase could not be determined.

Previous studies reported that L-arginine metabolism may influence regulatory T-cell (Treg) differentiation and immunometabolism [48]. Although no apparent impairment of MVST expansion or effector function was observed under the L-arginine–enriched condition, Treg-associated markers were not specifically evaluated in the present study. Therefore, the potential impact of L-arginine supplementation on Treg differentiation and function cannot be excluded and warrants future investigation.

## Conclusion

Supplementation of L-arginine enhances the durability of VSTs during *in vitro* manufacturing. Across expansion, antigenic restimulation, and cryopreservation and thawing, L-arginine consistently improved VST persistence, primarily through attenuation of apoptosis. Our data indicate that L-arginine availability modulates cellular responses to γc cytokines, with associated changes observed in signaling pathways, such as Akt and STAT5. Overall, these findings support L-arginine supplementation as a simple and translationally relevant strategy to improve the durability of VST products.

### Limitations

This study has several limitations. First, all experiments were performed using *in vitro*–expanded VSTs derived from healthy donors; therefore, the *in vivo* persistence and antiviral efficacy of L-arginine supplementation were not directly assessed. Second, although we observed enhanced survival and preserved MMP under the L-arginine–supplemented conditions, we did not perform direct metabolic flux analyses, such as measurements of glycolysis or oxidative phosphorylation, and thus cannot establish a definitive metabolic mechanism underlying these effects. In addition, we did not directly assess the intracellular or extracellular dynamics of L-arginine or its downstream metabolites, including how L-arginine availability is regulated and sensed during culture. Finally, potential effects on other immune cell types, including Tregs, antigen-driven T-cell activation, alternative arginine-dependent processes (e.g. autophagy), and long-term clonal composition, were not systematically evaluated and warrant further investigation.

## Supplementary Material

ltag014_Supplementary_Data

## Data Availability

The datasets generated during and/or analyzed during the current study are not publicly available but are available from the corresponding author upon reasonable request.

## Patent application

The underlying platform, which comprises several novel technologies, including L-arginine supplementation, is protected by an international patent application (PCT/JP2025/033758).
