# Supplementary material for: L-Arginine supplementation improves the survival of virus-specific CD8+ T cells during in vitro expansion
Source: Immunother Adv. 2026 Sep 16;6(1):ltag014. doi: 10.1093/immadv/ltag014 (PMC13580084; doi:10.1093/immadv/ltag014)

Supplemental Figure 5

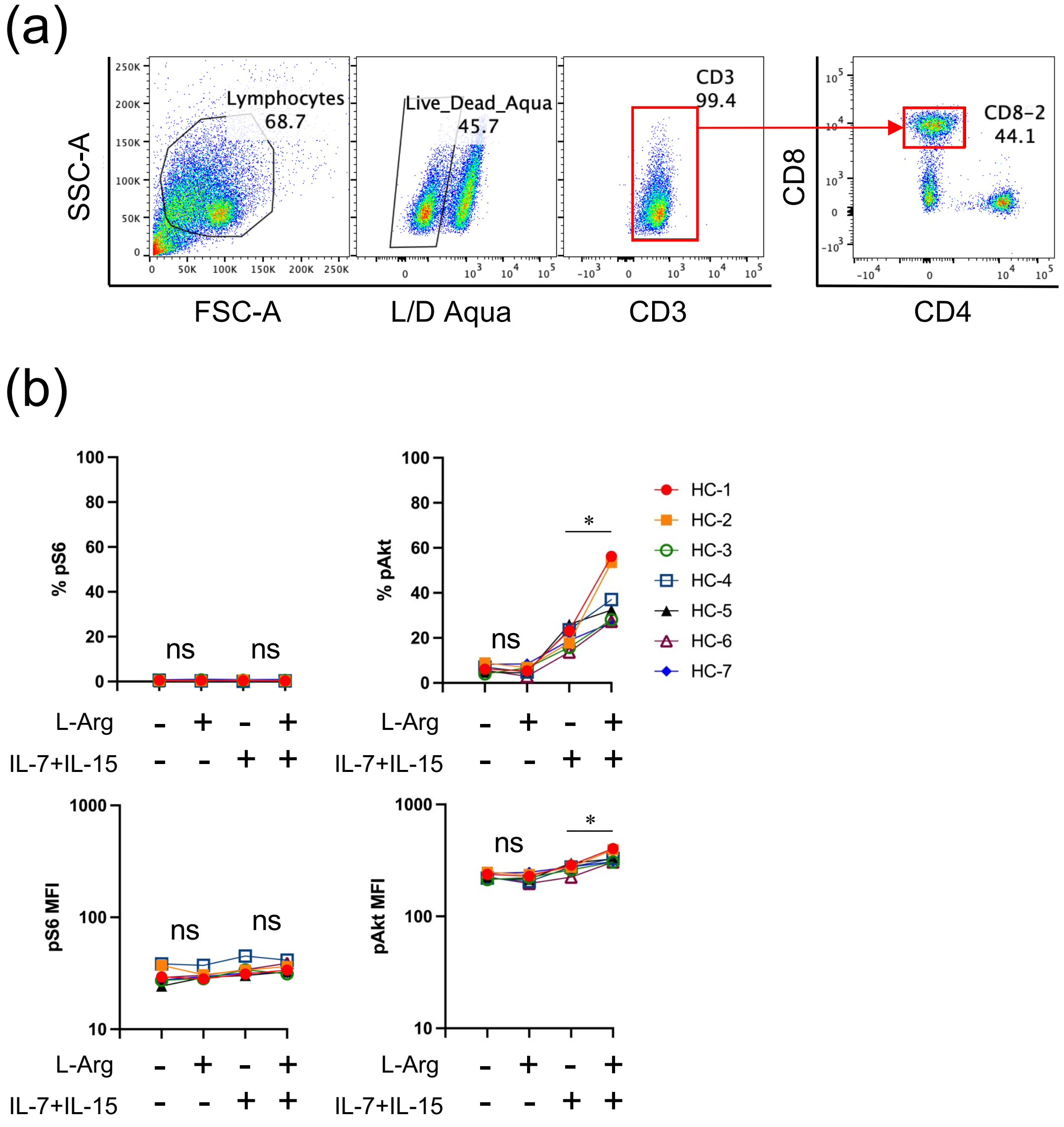

Supplement: Supplementary file 1 [file ltag014_supplementary_data.zip › FIgure S5.pdf]
