# Supplementary material for: L-Arginine supplementation improves the survival of virus-specific CD8+ T cells during in vitro expansion
Source: Immunother Adv. 2026 Sep 16;6(1):ltag014. doi: 10.1093/immadv/ltag014 (PMC13580084; doi:10.1093/immadv/ltag014)
Supplement: Supplementary file 1 [file ltag014_supplementary_data.zip › Supplementary table 1.docx]

**Supplementary Table S1. Antibodies and reagents used in this study**

| Target | Clone | Fluorophore | Vendor | Country |
| --- | --- | --- | --- | --- |
| pAkt (Ser473) | M89-61 | AF488 | BD Biosciences | California, USA |
| PD-1 | EH12.1 (EH12) | BB700 | BD Biosciences | California, USA |
| pS6 (Ser235/236) | N7-548 | PE | BD Biosciences | California, USA |
| Cleaved caspase-3 | C92-605 | V450 | BD Biosciences | California, USA |
| CCR7 | 2-L1-A | BV650 | BD Biosciences | California, USA |
| IFN-γ | 4S.B3 | BV786 | BD Biosciences | California, USA |
| CD3 | SK7 | BUV615 | BD Biosciences | California, USA |
| CD4 | SK3 | BUV496 | BD Biosciences | California, USA |
| CD8 | HIT8a | BUV563 | BD Biosciences | California, USA |
| CD27 | M-T271 | BUV661 | BD Biosciences | California, USA |
| CD28 | CD28.2 | BUV737 | BD Biosciences | California, USA |
| CD45RA | HI100 | BUV805 | BD Biosciences | California, USA |
| CD25 | BC96 | PE-Cy7 | BioLegend | California, USA |
| CD62L | DREG-56 | AF700 | BioLegend | California, USA |
| TIM-3 | F38-2E2 | APC-Cy7 | BioLegend | California, USA |
| BCL-2 | 100 | PE | BD Biosciences | California, USA |
| BCL-xL | 54H6 | PE-Cy7 | Cell Signaling Technology | Massachusetts, USA |
| MCL-1 | D2W9E | AF647 | Cell Signaling Technology | Massachusetts, USA |
| Annexin V | – | FITC | Invitrogen | Massachusetts, USA |
| CD3 | BW264/56 | APC | Miltenyi Biotec | Germany |
| CD4 | VIT4 | VioBlue | Miltenyi Biotec | Germany |
| CD8 | SFCI21Thy2D3 | PC7 | Beckman Coulter | California, USA |
| CD3 | HIT3a | Pacific Blue | BioLegend | California, USA |
| CD4 | OKT4 | FITC | BioLegend | California, USA |
| CD8a | HIT8a | PerCP | BioLegend | California, USA |
| CD107a | H4A3 | PE | BioLegend | CA, USA |
| IFN-γ | 4S.B3 | APC-Cy7 | BioLegend | California, USA |
| TNF-α | Mab11 | APC | BioLegend | CA, USA |
| pSTAT5 (Y694) | 47 | AF488 | BD Biosciences | California, USA |
| LIVE/DEAD Fixable Aqua Dead Cell Stain kit | – | – | Invitrogen | Massachusetts, USA |
| BD Horizon Fixable Viability Stain 575V | – | – | BD Biosciences | California, USA |
| CD28 antibody | 15E8 | – | Miltenyi Biotec | Germany |
| CD49d antibody | MZ18-24A9 | – | Miltenyi Biotec | Germany |
| GolgiStop | – | – | BD Biosciences | California, USA |
