# Supplementary material for: L-Arginine supplementation improves the survival of virus-specific CD8+ T cells during in vitro expansion
Source: Immunother Adv. 2026 Sep 16;6(1):ltag014. doi: 10.1093/immadv/ltag014 (PMC13580084; doi:10.1093/immadv/ltag014)
Supplement: Supplementary file 1 [file ltag014_supplementary_data.zip › Supplementary_figure_legends.docx]

**Supplementary Figure Legends**

**Supplementary Figure 1. Phenotypic and functional profiles of MVSTs under R10 conditions.**

Alt text: Gating strategy and phenotypic characterization of MVSTs under R10, including identification of CD3^+^, CD4^+^, CD8^+^, and virus-specific IFN-γ–producing cells.

(a) Gating strategy for CD3, CD8, CD4 expression, and intracellular IFN-γ analysis in MVSTs. After gating on lymphocytes and exclusion of doublets and dead cells, CD3^+^ T cells were identified, followed by analysis of CD8 and CD4 expression. CD8^+^ T cells were further gated, and expression of IFN-γ, TNF-α, and CD107a was assessed following stimulation with the corresponding viral peptide pools. (b) Frequencies of CD3, CD8, and CD4 expression in MVSTs. (c) Frequencies of total virus–specific CD8^+^ T cells and virus–specific CD8^+^ T cells reactive to CMV, EBV, HAdV, HHV-6, BKV, or JCV are shown. (d) Gating strategy used for Figure 1d. After gating on lymphocytes and exclusion of doublets, CD3^+^ T cells were identified. CD3^+^ T cells were analyzed for apoptosis by Annexin V and propidium iodide (PI) staining. Each dot represents an individual donor (biological replicate; n = 7).

**Supplementary Figure 2. Supplementary characterization and control analyses of MVSTs cultured under R10 and the L-arginine–enriched conditions.**

Alt text: Supplementary flow cytometry and characterization analyses of MVSTs cultured under R10 or L-arginine–enriched conditions, including proliferation, apoptosis gating strategy, absolute inhibitor–response analyses for MCL-1 and BCL-2 blockade, extracellular pH measurements, and D-arginine stereospecific control experiments.

(a) Fold change in cell proliferation between R10 and L-arginine–enriched conditions, measured using acridine orange–propidium iodide (AO/PI) staining. (b) Gating strategy used for Figure 2c. Lymphocytes were gated based on forward and side scatter, followed by exclusion of doublets and dead cells. CD3^+^ CD8^+^ T cells were then gated and analyzed for apoptosis by Annexin V and PI staining. (c) Absolute viable cell number of MVSTs cultured under R10 or L-arginine–enriched conditions in the presence of IL-7 and IL-15 with either the MCL-1 inhibitor AZD5991 (left) or BCL-2 inhibitor ABT-199 (right) for 24 h. (d) Absolute frequencies of Annexin V^-^ PI^-^ CD8^+^ T cells in MVSTs cultured under R10 or L-arginine–enriched conditions in the presence of IL-7 and IL-15 with either AZD5991 (left) or ABT-199 (right) for 24 h. (e) The pH of R10, D-arginine–supplemented medium, and L-arginine–enriched medium in the absence of cells was measured after incubation at 37°C with 5% CO_2_ for 3 h. (f) Comparative analysis of frequencies of viable cells (LIVE/DEAD Aqua–negative), CD3^bright^ T cells, and Annexin V^+^ cells in CD8^+^ T cells under R10, D-arginine supplemented, and L-arginine–enriched conditions. Each dot represents an individual donor (biological replicate; n = 7).

**Supplementary Figure 3. Gating strategies and additional phenotypic analyses of CD8^+^ T cells within MVSTs.**

Alt text: Flow cytometric gating strategies and representative plots, and additional phenotypic analyses used for evaluation of MMP, exhaustion markers, and differentiation phenotypes in CD8^+^ T cells cultured under R10 or L-arginine–enriched conditions.

(a) Gating strategy used for Figure 3a. Lymphocytes were gated based on forward and side scatter, followed by exclusion of doublets and dead cells. CD3^+^ CD8^+^ T cells were then gated and analyzed for mitochondrial membrane potential using MT-1 staining. (b) Gating strategy used for Figure 3b, 3c, and S3c. After gating on lymphocytes, doublets and dead cells were excluded. cCasp-3⁻ cells were then gated to exclude apoptotic cells, followed by identification of CD3⁺ T cells. CD3⁺ T cells were subsequently gated for CD8 expression, and CD8⁺ T cells were analyzed for the expression of exhaustion markers (PD-1 and TIM-3). Differentiation markers (CD27, CD28, and CD62L) were evaluated within the CCR7^-^ CD8^+^ T cell population. (c) Representative flow cytometric plots and the comparative analysis data of CD3, CD8, CD4, and differentiation markers (CCR7 and CD45RA) expression in CD8^+^ T cells under R10 and L-arginine–enriched conditions. CD8^+^ T cells were classified into naïve (CCR7^+^ CD45RA^+^), central memory (T_CM_; CCR7^+^ CD45RA^-^), effector memory (T_EM_; CCR7^-^ CD45RA^-^), and terminally differentiated effector memory re-expressing CD45RA (T_EMRA_; CCR7^-^ CD45RA^+^) subsets. Each dot represents an individual donor (biological replicate; n = 7).

**Supplementary Figure 4. Gating strategies and representative flow cytometric plots for phenotypic analysis of virus–specific CD8^+^ T cells.**

Alt text: Flow cytometric gating strategies and representative plots used for the analysis of apoptotic, anti–apoptotic, and exhaustion markers in virus–specific CD8^+^ T cells cultured under R10 or L-arginine–enriched conditions.

(a) Representative flow cytometric plots showing expression of apoptotic markers (Annexin V and cCasp-3) and anti–apoptotic markers (MCL-1 and BCL-2) in virus–specific CD8^+^ T cells. (b) Gating strategy used for Figure 4c. After gating on lymphocytes, doublets and dead cells were excluded. cCasp-3⁻ cells were then gated to exclude apoptotic cells, followed by identification of CD3⁺ T cells. CD3⁺ T cells were subsequently gated for CD8⁺ cells, and IFN-γ⁺ populations were identified. CD8⁺ IFN-γ⁺ T cells were then analyzed for the expression of exhaustion markers. (c) Representative flow cytometric plots showing expression of exhaustion markers in virus–specific CD8^+^ T cells.

**Supplementary Figure 5. Additional analyses of Akt and S6 phosphorylation in MVSTs.**

Alt text: Flow cytometry analyses of Akt and S6 phosphorylation in CD8^+^ T cells within MVSTs, including gating strategies and additional analyses following IL-7 and IL-15 stimulation in the presence or absence of L-arginine.

(a) Gating strategy used for Figures 6a, 6b, and S6b–c. Lymphocytes were gated, followed by exclusion of dead cells. CD3^+^ CD8^+^ T cells were then gated and analyzed for phosphorylation of Akt, S6, and STAT5. (b) Additional analysis of phosphorylated S6 and Akt in CD8^+^ T cells at 30 min following IL-7 and IL-15 stimulation in the presence or absence of L-arginine. Each dot represents an individual donor (biological replicate; n = 7).

**Supplementary Figure 6. Additional analyses of STAT5 signalling and pharmacological inhibition in MVSTs.**

Alt text: Supplementary flow cytometric and pharmacological analyses of MVSTs, including comparison of CD25 expression under R10 or L-arginine–enriched conditions, cytokine–induced STAT5 phosphorylation in the presence or absence of L-arginine, short–term L-arginine deprivation experiments, and inhibitor studies targeting mTORC1/2 and JAK/STAT signalling pathways.

(a) Representative flow cytometric plots and frequencies of CD25^+^ CD8^+^ T cells within MVSTs maintained under R10 and L-arginine–enriched conditions. (b) Frequencies of phosphorylated STAT5 in CD8^+^ T cells at 30 min following stimulation with IL-2 in the presence or absence of L-arginine. (c) Frequencies of phosphorylated STAT5 in CD8^+^ T cells at 60 min following stimulation with IL-2, IL-7, or IL-15 in the presence or absence of L-arginine. (d) After 30 min of L-arginine deprivation, the frequency of Annexin V^-^ CD8^+^ T cells and MT-1 expression (frequency and MFI) within Annexin V^-^ CD8^+^ T cells were analyzed by flow cytometry. (e) Viable cell number analysis of MVSTs cultured with or without L-arginine under IL-7 and IL-15 stimulation in the presence of sapanisertib (dual mTORC1/2 inhibitor), tofacitinib (JAK inhibitor), or the combination of both inhibitors for 48 h. Each dot represents an individual donor (biological replicate; n = 7).
